# Wide substrate range for a candidate bioremediation enzyme isolated from *Nocardioides* sp. strain SG-4 G

**DOI:** 10.1093/femsle/fnad085

**Published:** 2023-09-02

**Authors:** Kishore K Krishnani, John G Oakeshott, Gunjan Pandey

**Affiliations:** CSIRO Environment, Canberra, ACT 2601, Australia; Central Institute of Fisheries Education, Versova, Andheri (West), Mumbai 400061, India; CSIRO Environment, Canberra, ACT 2601, Australia; Applied BioSciences, Macquarie University, North Ryde, New South Wales 2113, Australia; CSIRO Environment, Canberra, ACT 2601, Australia

**Keywords:** plasticizers, herbicides, bioremediation, biodegradation, substrate specificity, enzyme

## Abstract

Narrow substrate ranges can impact heavily on the range of applications and hence commercial viability of candidate bioremediation enzymes. Here we show that an ester hydrolase from *Nocardioides* strain SG-4 G has potential as a bioremediation agent against various pollutants that can be detoxified by hydrolytic cleavage of some carboxylester, carbamate, or amide linkages. Previously we showed that a radiation-killed, freeze-dried preparation (ZimA) of this strain can rapidly degrade the benzimidazole fungicide carbendazim due to the activity of a specific ester hydrolase, MheI. Here, we report that ZimA also has substantial hydrolytic activity against phthalate diesters (dimethyl, dibutyl, and dioctyl phthalate), anilide (propanil and monalide), and carbamate ester (chlorpropham) herbicides under laboratory conditions. The reaction products are substantially less toxic, or inactive as herbicides, than the parent compounds. Tests of strain SG-4 G and *Escherichia coli* expressing MheI found they were also able to hydrolyse dimethyl phthalate, propanil, and chlorpropham, indicating that MheI is principally responsible for the above activities.

## Teaser

A soil bacterium exhibits bioremediation potential by degrading pollutants. ZimA, an enzyme preparation from this strain, effectively breaks down fungicides and herbicides, reducing their toxicity.

## Introduction

Enzymatic bioremediation is attracting increasing attention as an effective, ‘green’ method for pollution abatement (Sutherland et al. 2004, Scott et al. 2008, Sharma et al. 2018, Thakur et al. 2019, Wackett and Robinson 2020, Mousavi et al. 2021, Saravanan et al. 2021). It generally involves the use of cell-free preparations of redox cofactor-independent enzymes as catalysts for rapid removal of organic and inorganic pollutants from contaminated environments (Scott et al. 2008, Thatoi et al. 2014, Sharma et al. 2018, Mousavi et al. 2021). Such enzymes have been developed for agricultural and industrial pollutants such as pesticides (Sutherland et al. 2004, Pandey et al. 2010, Coppin et al. 2012, Hennessy et al. 2018), plastics/plasticizers (Boll et al. 2020), and explosives (Fida et al. 2014, Karthikeyan et al. 2016).

However, very few of these enzymes have yet seen widespread commercial use, despite demonstrated efficacy in specific field trials, and in many cases this has been because their substrate range has been too narrow to allow versatile use against a range of pollutants (Sutherland et al. 2004, Scott et al. 2008, Scott et al. 2010). Many pollutants can be degraded in cofactor-independent reactions catalysed by ester hydrolases, but the diversity of ester bonds and substituent moieties has so far hindered widespread deployment of such enzymes as bioremediation agents (Scott et al. 2008, Thakur et al. 2019). Modern protein engineering technology now often enables a broadening of substrate range in respect of the substituent moieties, but enzymes able to attack different types of ester bonds have generally proven elusive. For example, several carboxylesterases can hydrolytically detoxify pyrethroid insecticides, but none are yet known to also hydrolyse organophosphates, carbamates, or amides (Hatfield and Potter 2011). Conversely many amidases and carbamate hydrolases have broad substrate specificity within amides and carbamates, respectively, but negligible activity for other substrate classes (Wu et al. 2020, Mishra et al. 2021). In a similar vein, the esterases so far known to degrade phthalate diesters do not degrade carbamates, amides, organophosphates, and other classes of ester pollutants (Boll et al. 2020).

One promising bioremediation enzyme in this respect is the ester hydrolase MheI from *Nocardioides* sp. strain SG-4 G. This ca. 25 kDa enzyme (awaiting an E.C. number from the Enzyme Commission) can be produced at scale, formulated in active form in a radiation-killed freeze-dried preparation of the wild type bacterium (hereafter designated ZimA, the term MheI being reserved for the enzyme itself), and used to decontaminate water from commercial fruit-dipping operations containing high levels of the benzimidazole fungicide carbendazim (methyl-1H-benzimidazol-2-ylcarbamate; MBC) (Pandey et al. 2010). Furthermore, carbendazim is a carbamate ester, but Mhe1 also hydrolyses model carboxylesters such as naphthyl acetate, *p-*nitrophenyl acetate, and methyl salicylate (Pandey et al. 2010).

Here we explore the potential of the MheI enzyme and ZimA formulation of it to hydrolyse and thereby detoxify some other widespread pollutants with ester, carbamate, or amide bonds. The compounds tested include three phthalate ester industrial pollutants (dimethyl, dibutyl, and dioctyl phthalate), an oxime carbamate insecticide (methomyl), and nine herbicides, specifically two anilides (propanil and monalide), one carbamate ester (chlorpropham), one triazolone (carfentrazone-ethyl), three sulfonylureas (metsulfuron-methyl, pyrazosulfuron-ethyl, and sulfosulfuron), an imidazolinone (imazethapyr), and a dintroaniline (pendimethalin).

## Material and methods

### Chemicals

Analytical grade dimethyl phthalate, monomethyl phthalate, dibutyl phthalate, dioctyl phthalate, propanil, monalide, chlorpropham, carfentrazone-ethyl, methomyl, 3-chloroaniline, 4-chloroaniline, and 3,4-dichloroaniline were purchased from Sigma-Aldrich (Castle Hill, Australia). Sulfosulfuron, metsulfuron-methyl, pyrazosulfuron-ethyl, imazethpyr, and pendimethalin were gifted by Dow Agrosciences (India). All other chemicals were used at the highest purity available commercially.

### Microorganisms and growth media

Isolation of *Nocardioides* sp. strain SG-4 G (V07/015 486, National Measurement Institute, Australia), generation of the *mheI* expressing vector pDEST17-mheI, and preparation of the ZimA extract were as described previously (Pandey et al. 2010). Strain SG-4 G, ZimA, and *E. coli* BL21-AI™ were obtained from our laboratory stocks.

Tests of the ability of strain SG-4 G to grow by utilizing some compounds as carbon sources used a previously described carbon-free minimal salt medium (MSM; Pandey et al. 2010), as did tests of the ability of pre-grown cells of this strain and *E. coli* pDEST17-mheI to degrade these compounds in the absence of cell growth. Powdered forms of the compounds were dissolved in MSM, which was then filtered through 0.22 µm sterilized filters (Millipore, MA). Nutrient agar (NA) and nutrient (NB) or Luria-Bertani (LB) broth were also used as rich media to support bacterial growth when required.

### Types of degradation assay

Four different types of assays were used to characterize the ability of ZimA to degrade the chemicals of interest. One simply used the radiation-killed freeze-dried ZimA preparation. The second involved growth assays to determine whether strain SG-4 G could use the test chemicals as a sole source of carbon and energy. The third tested pre-grown strain SG-4 G cells to see whether their ability to hydrolyse the test chemicals was constitutively expressed. Finally, pre-grown *E. coli* cells expressing the *mheI* gene were tested to see if the MheI enzyme was responsible for the hydrolysis of the test chemicals. Except where noted, all assays were carried out in triplicate.

### Degradation assays with ZimA

Following Pandey et al. (2010), various doses of ZimA were added to 50 ml MSM in 100 ml Erlenmeyer flasks containing the test chemicals (at concentrations below their aqueous solubility limits), and the reactions allowed to proceed under aseptic conditions (28°C, 100 rpm). One-millilitre samples were collected at various time points and filtered through 0.45 µm sterile filters (Millipore, MA), before formic acid was added to a final concentration of 1% v/v. Samples were then stored at 4°C until analysed as below. Flasks with all reagents except ZimA were used as negative controls.

### Degradation assays with growing cells of strain SG-4 G

An aerobically grown (28°C, 100 rpm) overnight seed culture of strain SG-4 G in NB was used to inoculate (1%, v/v) 50 ml of the supplemented MSM, which was then incubated at 28°C with shaking at 100 rpm. One-millilitre samples were collected at various time points, filtered through 0.45 µm filters (Millipore, MA), formic acid added to a final concentration of 1% v/v, and the resultant mixture stored at 4°C until analysed. Flasks without SG-4 G cells but with the respective chemicals of interest were used as negative controls.

### Degradation assays with pre-grown cells of strain SG-4 G and *E. coli* expressing *mheI*

Following Pandey et al. (2010), a single colony of SG-4 G or *E. coli* BL21-AI™ pDEST17-mheI was picked from a plate and inoculated into 1 l of nutrient broth (NB) for SG-4 G or 250 ml of LB plus 100 mg/ml ampicillin for *E. coli* pDEST17-mheI. These cultures were then incubated at 28°C with shaking at 100 rpm until they reached mid exponential phase (OD_600_ ∼0.6 and 1.0, respectively). Fifty millilitre of NB-grown cells and 5 ml of LB-grown cells were then harvested, washed with ice-cold MM, and resuspended in 10 ml of MM containing 10 mM glucose and the requisite concentration of the compound in question. The resuspended cultures were then incubated at 28°C with shaking at 100 rpm. Samples were collected at various time points, filtered through 0.45 µm filters (Millipore), formic acid added to a final concentration of 1% v/v, and the resultant mixture stored at 4°C until analysed. Samples containing all the reagents except the pre-grown cells were used as negative controls.

### Analytical methods

A high-performance liquid chromatography system (HPLC, Agilent Technologies, CA) coupled in series to either a diode array detector (DAD; Agilent Technologies) or a time-of-flight mass spectrometer (TOF/MS; Agilent Technologies) was used for quantitative and qualitative analysis, respectively.

Compounds (20 µl injection per sample) were separated and eluted at 25°C on an Aqua C18 column (5 µm particle size, 250 × 4.60 mm; Phenomenex, CA) under various isocratic conditions of acetonitrile in water (containing formic acid at a final concentration of 0.1%) as a mobile phase at various flow rates (Supplementary Table S1). Dimethyl phthalate, propanil, monalide, and chlorpropham and their degradation products (monomethyl phthalate, 3,4-dichloroaniline, 4-chloroaniline, and 3-chloroaniline, respectively) were quantified using authentic standards with the DAD operating at appropriate wavelengths (Supplementary Table S1). The identities of all test chemicals and, where possible, their transformation products were confirmed with the TOF/MS operating as described previously (Pandey et al. 2010).

## Results

### Phthalate esters

**Figure 1.**
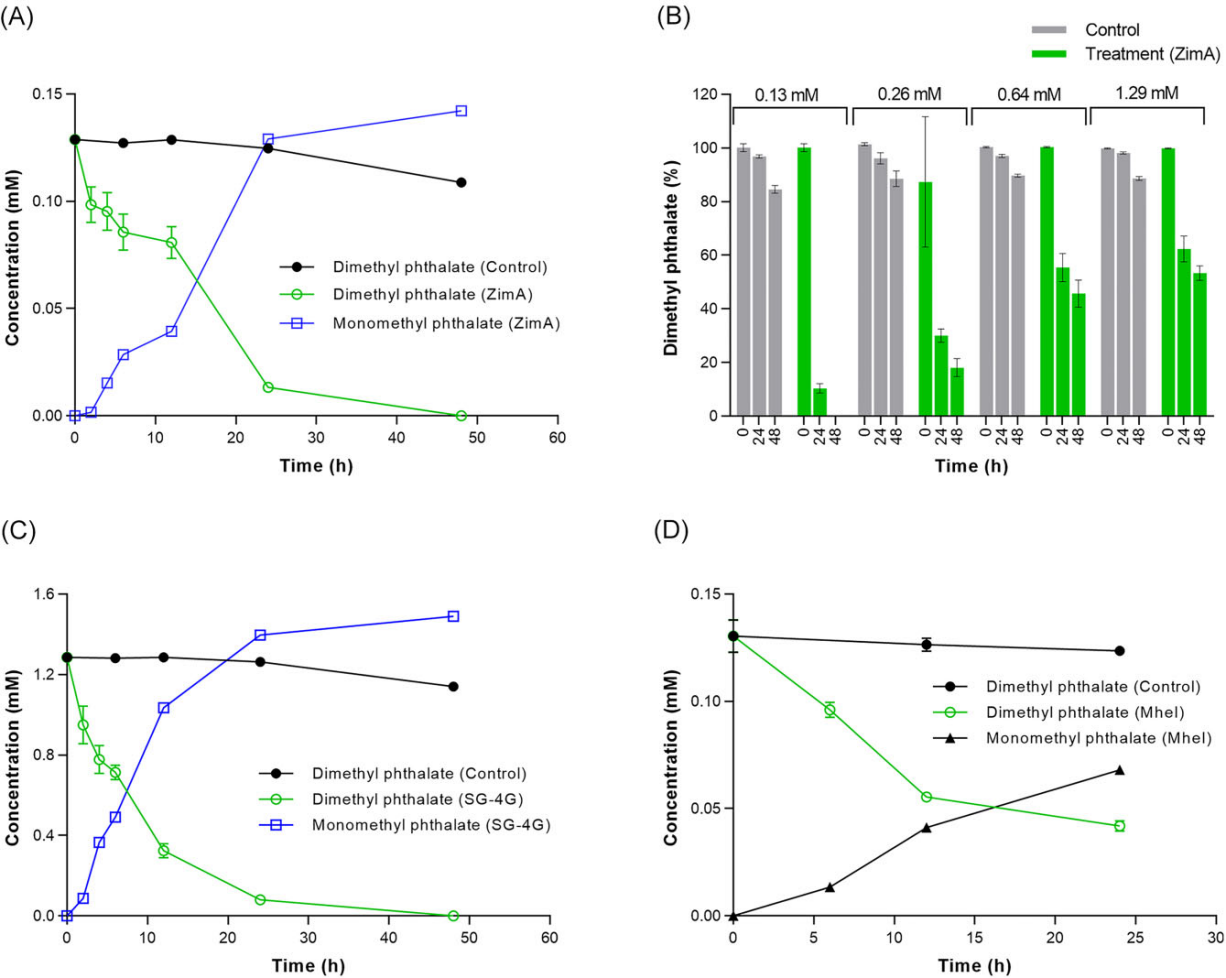
Degradation of dimethyl phthalate by ZimA, strain SG-4 G, and *E. coli* cells expressing the *mhe*I gene: (A) Degradation of 0.130 mM dimethyl phthalate and production of monomethyl phthalate by 400 ppm ZimA; (B) Degradation of different concentrations of dimethyl phthalate by 400 ppm ZimA; (C) Degradation of 1.28 mM dimethyl phthalate by live cells of the strain SG-4 G; and (D) Degradation of 0.13 mM dimethyl phthalate by *E. coli* cells expressing *mhe*I.

**Figure 2.**
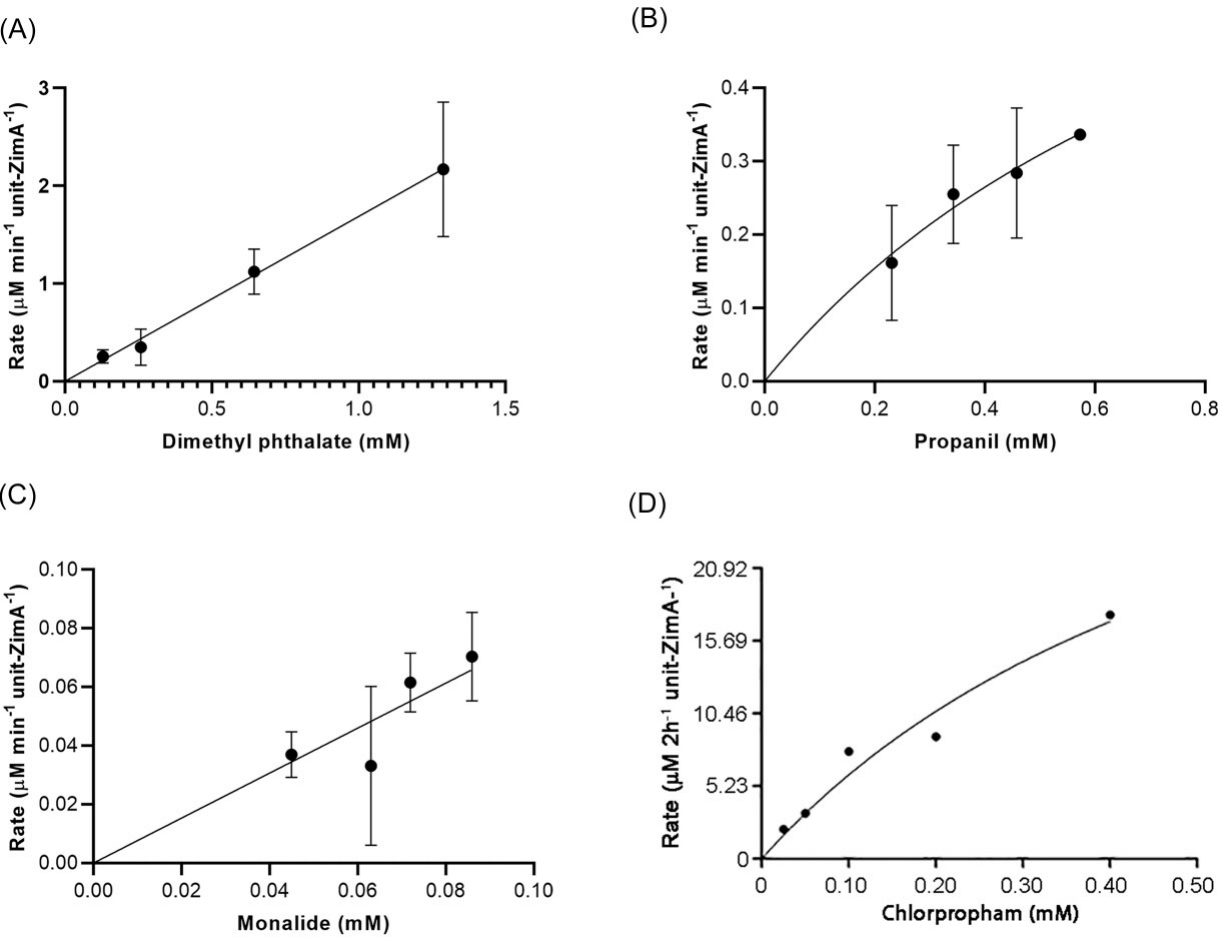
Plots of degradation rates against substrate concentrations for the reactions of ZimA with (A) dimethyl phthalate, (B) propanil, (C) monalide, and (D) chlorpropham.

ZimA at 400 ppm completely transformed 130 µM dimethyl phthalate within 48 h. The reaction was approximately linear over the first 24 h, after which time over 90% of substrate was consumed (Fig. 1A and Supplementary Fig. S1). Assays starting with higher substrate concentrations (maximum 1.29 mM) did not increase the absolute amount of substrate consumed, so the percentage consumed decreased (Figs 1B and 2A). This indicates that the *K*_M_ of the enzyme(s) catalysing the first cleavage reaction was <130 µM. Near-stoichiometric amounts of the product, monomethyl phthalate (Rt. 7.53 min, *m*/*z* 181) were observed (Fig. 1C), so only one of the two ester bonds of the phthalate was efficiently hydrolysed. However, small amounts of phthalic acid in its protonated anhydride form (*m*/*z* 149) were detected, suggesting some cleavage of the remaining ester bond (data not shown).

Tests for growth of strain SG-4 G on MM supplemented with dimethyl phthalate found that it was not used as a sole source of carbon and energy for growth (data not shown). However, pre-grown live SG-4 G cells harvested from 50 ml cultures (OD_600_ ∼ 0.6) completely transformed 1.29 mM dimethyl phthalate in 24 h (Fig. 1C), while pre-grown *E. coli* cells harvested from 5 ml cultures (OD_600_ ∼ 1) expressing a his-tagged version of MheI transformed just over half (68%) the 130 µM dimethyl phthalate provided in the same timeframe (Fig. 1D). As with ZimA, both these cell types produced near-stoichiometric amounts of monomethyl phthalate (Fig. 1C and D). We do not know the relative concentrations of the MheI in the two cell types, or whether the his-tag influenced activity. However, the low yield of Mhe1 obtained from the *E. coli* cells (Pandey et al. 2010) suggests MheI was at least a major contributor to the phthalate degradation activity of ZimA.

**Figure 3.**
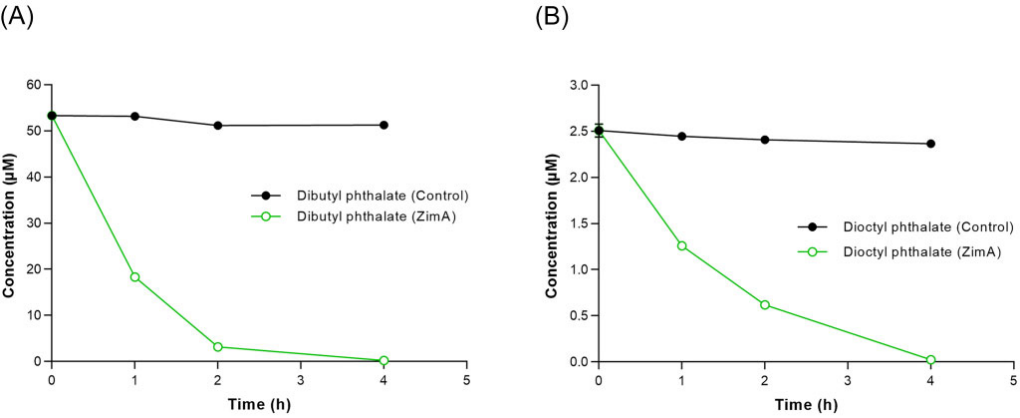
Degradation of 54 μM dibutyl phthalate (A) and 26 μM dioctyl phthalate (B) by 400 ppm ZimA.

The much lower aqueous solubilities of dibutyl and dioctyl phthalate (~40.2 and 0.56 µM, respectively) meant equivalent time course analyses of their degradation by 400 ppm ZimA had to use much lower starting substrate concentrations. Nevertheless, these assays showed complete degradation of 54 and 2.56 µM of those substrates, respectively, within 4 h (Fig. 3). The respective products, monobutyl phthalate and monooctyl phthalate, were detected at their expected *m*/*z* values (223 and 279, respectively; Supplementary Figs S2 and S3) but could not be quantified because authentic standards were not available. Only trace amounts of phthalic acid were seen (data not shown).

### Anilide herbicides

**Figure 4.**
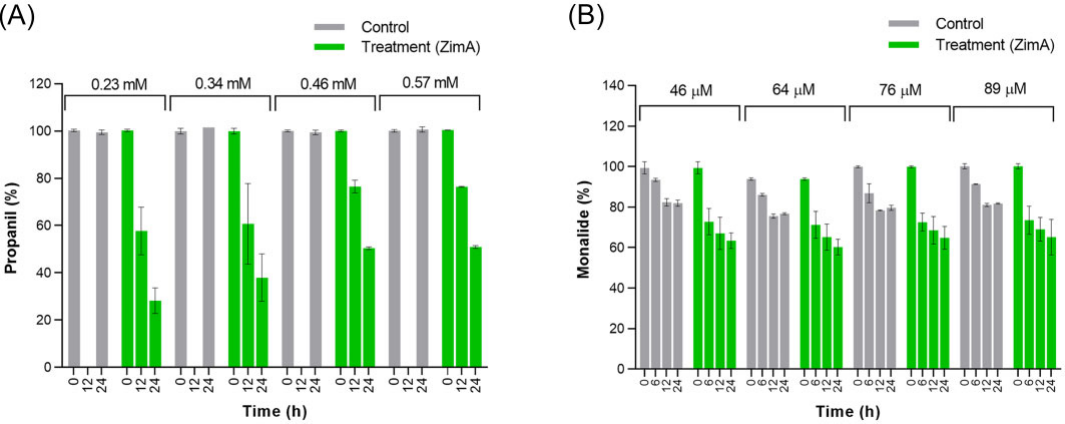
Degradation of different concentrations of propanil (A) and monalide (B) by 800 ppm ZimA.

Assays of propanil (*N*-(3,4-dichlorophenyl)propanamide) at starting concentrations between 0.23 and 0.57 mM with 800 ppm ZimA showed 50%–70% of the substrate, depending on its starting concentration, were degraded within 24 h (Figs 2B, 4A, and Supplementary Fig. S4). As with dimethyl phthalate, the percentage degraded was lower with the higher starting concentration but, unlike the dimethyl phthalate situation, the absolute amount was higher. This suggests the *K*_M_ for propanil could be as high or higher than the highest starting concentration (0.57 mM). As with some of the other substrates tested, the low aqueous solubility limit of propanil (0.6–1.0 mM) constrained our ability to further investigate the substrate concentration dependence of the reaction.

The incomplete degradation of the propanil also enabled us to compare the amounts degraded in the first and second 12 h of the assays. Only ~20% less was degraded in the second interval than the first (Supplementary Fig. S5). Thus, under the conditions of the assay at least, the ZimA preparation should be sufficiently stable to continue significant degradative activity for several days.

The metabolite expected from propanil hydrolysis, 3,4-dichloroaniline, was detected in these assays (Rt. 6.39 min, *m*/*z* 162), but in less than stoichiometric amounts, just 0.08–0.15 mM at 24 h (data not shown). This was probably not due to further metabolism because the transformations known for this compound require cofactors, which the cell-free ZimA preparation would have limited ability to recycle (Scott et al. 2008). Rather we expect that the higher hydrophobicity of the 3,4-dichloroaniline may have compromised its recovery during sample processing.

As with dimethyl phthalate, tests for growth of strain SG-4 G on MM supplemented with propanil found it was not used as a sole source of carbon and energy for growth (data not shown). However, the pre-grown SG-4 G cells degraded 0.272 mM propanil completely after 18 h, with 0.10 mM 3,4-dichloroaniline recovered, and qualitatively similar results were obtained for *E. coli* cells expressing MheI (data not shown).

Our monalide (*N-*(4-chlorophenyl)-2,2-dimethylpentanamide) preparation contained 10% (based on peak area) of an impurity that the supplier termed an isomer. We suspect this was a structural isomer of monalide, possibly *N*-(2-chlorophenyl)-2,2-dimethylpentanamide or *N*-(3-chlorophenyl)-2,2-dimethylpentanamide, since no geometric or stereoisomers of it are possible. Reactions of 800 ppm ZimA with four starting monalide concentrations between 46 and 89 µM showed progressive degradation over 24 h (Figs 2C, 4B, and Supplementary Fig. S6). Up to 35%–40% of both the nominate monalide and the isomer were degraded in this time, although this was only 10%–15% above background degradation in the no-enzyme negative controls (Supplementary Fig. S7). There was little difference between starting concentrations in the amount of monalide degraded in percentage terms, but more in terms of absolute amounts was seen at the highest starting concentration, suggesting *K*_M_ could be as high or higher than 89 µM. As with propanil, there was less than stoichiometric recovery (<5%) of the hydrolytic product, in this case 4-chloroaniline (Rt. 4.33 min, *m*/*z* 128; Supplementary Fig. S7), again, we suspect, reflecting losses due to its high hydrophobicity during sample processing.

Consistent with the results for propanil, strain SG-4 G was unable to grow on MM supplemented with monalide, but pre-grown SG-4 G cells degraded over 90% of 19 µM of it in 18 h, forming 3 µM 4-chloroaniline (data not shown).

### Chlorpropham

**Figure 5.**
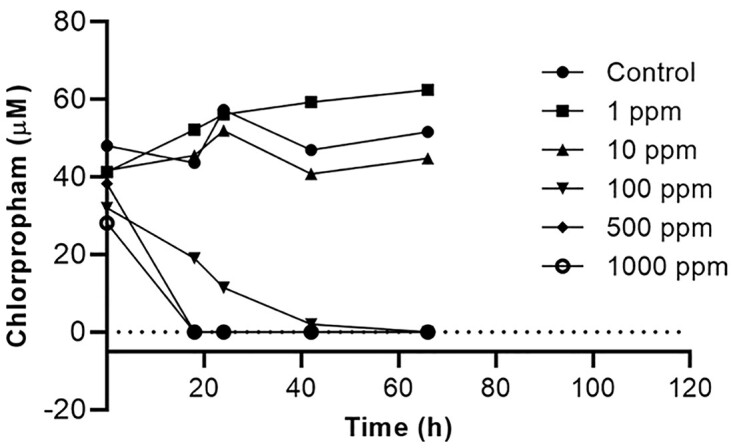
Degradation of 46.8 μM chlorpropham by varying concentrations ZimA.

Five doses of ZimA varying from 1 to 1000 ppm were tested for their ability to degrade 42 μM chlorpropham (Fig. 5 and Supplementary Fig. S8). There was no detectable degradation, even at 96 h, with 1 or 10 ppm ZimA, but complete degradation at 18 h with 500 and 1000 ppm, and largely complete degradation at 42 h with 100 ppm. Interestingly, in the latter case, significant degradation continued between 24 and 42 h, confirming the stability of the enzyme evidenced in the propanil results above. One product expected, 3-chloroaniline (Rt. 5.5 min, *m*/*z* 128) (the other being CO_2_), was detected at the higher enzyme doses but, as with the chloro- and dichloroanilines produced from monalide and propanil, respectively, at less than stoichiometric amounts and, in this case, there was too little to quantitate accurately (Supplementary Fig. S8).

Assays at various substrate concentrations showed the activity/substrate concentration curve had not plateaued at the highest substrate concentration (37 μM), suggesting the *K*_M_ may exceed the aqueous solubility limit (42 μM; ICSC 2004) of the chlorpropham (Fig. 2D).

As with the other compounds above, strain SG-4 G did not grow on MM supplemented with 40 μM chlorpropham but pre-grown SG-4 G cells and *E. coli* cells expressing MheI could transform it to the same product as ZimA, in this case 3-chloroaniline (data not shown).

### Other compounds

Four hundred ppm ZimA was also incubated for 24 h with six other herbicides and an insecticide at concentrations approaching their respective aqueous solubilities. LC-MS/TOF analysis showed complete degradation of 24–48 µM of the triazolone herbicide carfentrazone-ethyl (Rt. 6.6, *m*/*z* 412) to carfentrazone (Rt. 3.6, *m*/*z* 384) and 24–131 µM of two sulfonylureas with carboxylester bonds, metsulfuron-methyl (Rt. 5.5 min, *m*/*z* 382, 131 µM), and pyrazosulfuron-ethyl (Rt. 5.4 min, *m*/*z* 415, 24 µM), to metsulfuron (Rt. 3.9 min, *m*/*z* 368), respectively (Supplementary Figs S9 and S10). However, the products of these reactions are also herbicidal, so these activities were not pursued further. No degradation was detected for a sulfonylurea without a carboxylester group (sulfosulfuron, 0.286 mM), or the imidazolinone (imazethapyr, 0.17 mM), dintroaniline (pendimethalin, 1.0 µM), or oxime carbamate (methomyl, 0.31, 0.77, 1.54 mM) (data not shown).

## Discussion

We have found that a radiation-killed freeze-dried preparation of a growth culture of strain SG-4 G can degrade three phthalate diesters, two anilide herbicides, and a carbamate ester herbicide in addition to its previously demonstrated activity for the benzimidazole fungicide carbendazim. Furthermore, we have shown that substantial amounts of these activities are due to the cofactor-independent MheI enzyme. Thus, the strain and enzyme have activity against carboxylester, carbamate, and amide linkages. Importantly, the hydrolysis of the phthalates greatly reduces their toxicity, and the hydrolysis of the carbendazim, anilides, and chlorpropham eliminates their respective fungicidal and herbicidal activities (Jonsson and Baun 2003, Pandey et al. 2009, Carvalho et al. 2010, Pujar et al. 2018). Given the ubiquity of the phthalates in various industries and the large numbers of benzimidazole fungicides and anilide and carbamate herbicides used in agriculture, strain SG-4 G and MheI are potentially versatile bioremediant resources.

Several other bacteria are known to degrade phthalate esters, and others are known to degrade benzimidazole, or other carbamate, anilide or other amide herbicides/pesticides (Xu et al. 2007, Liang et al. 2008, Benjamin et al. 2015, Boll et al. 2020, Wu et al. 2020; Supplementary Table S2). In a few cases, the enzyme responsible for the degradation has also been characterized (Supplementary Table S2) (Zhang et al. 2012, 2019, Li et al. 2013, Sun et al. 2013, Chen et al. 2016).

**Figure 6.**
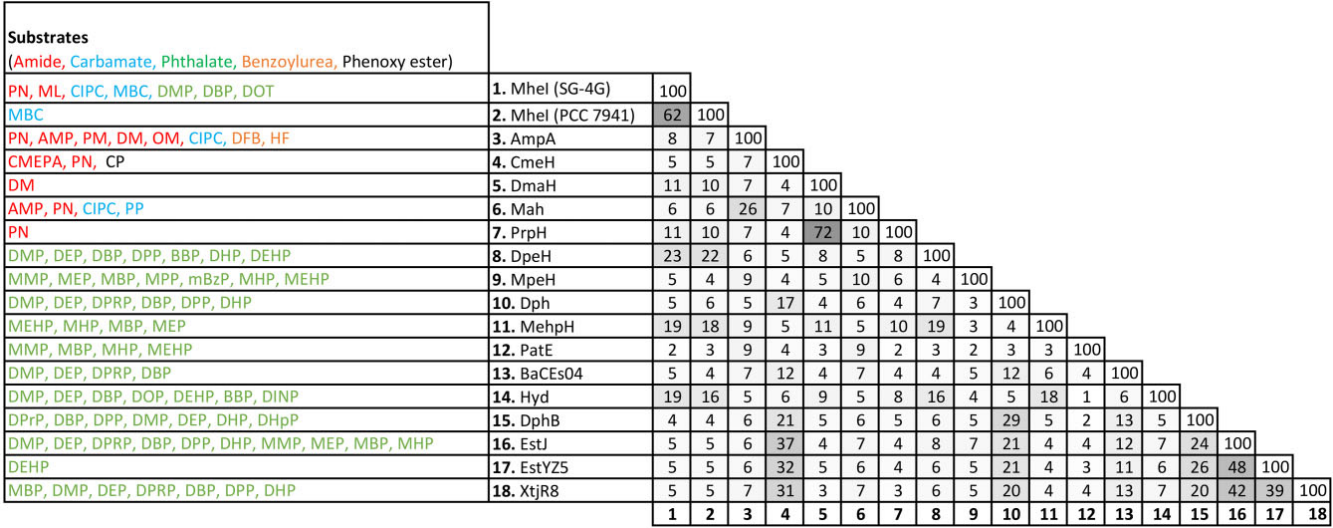
Heat map-coded correlation matrix of amino acid similarity percentages for enzymes with activity against any of the phthalates, carbamates, amides found to be substrates for MheI here or in Pandey et al. (2010). Note that only one of the four MheI isolates other than the one herein from SG-4 G is shown because the rest were not biochemically characterized in detail. Abbreviations for substrates and accession numbers for enzymes are as per Supplementary Table S2. Clustal Omega 1.2.2 (mBed algorithm) was used for multiple protein sequence alignment and generating the identity matrix (Sievers and Higgins 2018).

Notwithstanding the well-known promiscuity of esterases for a range of substrates within particular chemotypes of pesticides (Kourist et al. 2008, Martinez-Martinez et al. 2018), however, none of these other bacteria/enzymes have yet been shown to possess both the carboxylesterase activity against the phthalates and carbamate hydrolase and/or amidase [and in one case also urease (Reichel et al. 1991)] activities against the herbicides/pesticides (Supplementary Table S2). On current knowledge at least, SG-4 G/MheI is thus uniquely versatile in showing both sets of activities. Notably three gene/enzyme systems (GenBank accession numbers CCI05716, ARQ80492, and AEA07594) closely related to *mheI*/MheI and two others (AEB78730 and AOW37542) that are identical to it have also been isolated from various soil bacteria and, if tested, these would likely also show a similar substrate range (Fig. 6) (Zhang et al. 2013, 2017).

Some of the other enzymes shown to have either the phthalate esterase or the herbicide/pesticide carbamate hydrolase or amidase/urease activities might also prove to have similarly wide substrate ranges if their individual substrate ranges were tested. Significantly, one, CemH, has shown amidase activity against anilide herbicides plus esterase activity against a phenoxyester insecticide (Supplementary Table S2) (Li et al. 2013). All these other enzymes, including CemH, show <20% similarity to ZimA and most also show <20% similarity to each other (Fig. 6). The exceptions in respect of the latter are the amidases, PrpH and DmhA, with 72% similarity to one another, and three enzymes isolated from metagenomes with phthalate esterase activity, which are up to 48% similar to one another and 31%–37% similar to Cemh. There are thus several largely independent lineages of enzyme to assess as potential broad-substrate-range bioremediation enzymes.

Significantly also, some of these lineages likely utilize different catalytic mechanisms, which could in turn impact on their ester or amide substrate ranges. For example, MheI and some other hydrolases utilize a charge relay mechanism that operates most effectively at neutral-basic pHs (Lei et al. 2017, Du et al. 2021), but the negatively charged carboxylic group generated by hydrolysis of the first ester bond in phthalate diesters is detrimental to hydrolysis of the second, for which more acidic pH is optimum 5.5–6.5 (Fojan 2000, Chahinian and Sarda 2009). This likely explains why ZimA/MheI is better able to hydrolyse the first than the second ester bond in these compounds. However, other esterases have been described that can effectively hydrolyse both the diesters and monoesters, and other individual bacteria and consortia are known that utilize two different esterases for the two reactions (Hara et al. 2010, Wu et al. 2013, Prasad and Suresh 2015, Lu et al. 2020, Qiu et al. 2020).

The limited data presently available also suggest that, steric constraints aside, the Mhe1 enzyme might prefer carboxylester and carbamate over amide (and urea) substrates. Combined across our study and Pandey et al. (2010), the highest rates calculated for ZimA with carbamates involved essentially complete degradation of 2.6 mM carbendazim in 24 h by 800 ppm enzyme and 47 µM chlorpropham in 18 h by 500 ppm, while that for a carboxylester was 130 µM dimethyl phthalate in 48 h by 500 ppm. By comparison, the highest value calculated for an amide involved about half of 23–57 µM propanil in 12 h by 800 ppm. Depending on the specifics of the application, substantial increases in the latter rates, in particular, would be needed for cost-effective bioremediation (see below). However, such increases should be achievable with modern enzyme engineering technology (Turner 2003, Lutz and Iamurri 2018, Sharma et al. 2018, Mousavi et al. 2021). Notably, while ZimA is already relatively stable in both laboratory and field environments, enzyme stability can also be enhanced with this technology (Pongsupasa et al. 2022, Teufl et al. 2022).

The two major approaches to bioremediation in which ZimA/MheI may be useful are the clean-up of contaminated water by free-enzyme formulations, as per ZimA, and the clean-up of contaminated soils by transgenic phytoremediation crops expressing MheI. The fact that SG-4 G expressed MheI constitutively (Pandey et al. 2010) is advantageous for the free enzyme applications because it facilitates its production. The fact that it had little activity for phthalate monoesters is disadvantageous for free enzyme remediation of the phthalates, although hydrolysis of the first ester bond still represents substantial detoxification (Jonsson and Baun 2003, Xu et al. 2016, Tian et al. 2019). Similarly, while the ZimA assays and SG-4 G growth assays suggest they do not completely mineralize the anilide herbicides, this need not be problematic as the hydrolysis destroys their herbicidal activity (Kanawai et al. 2016). Some of the herbicide-deactivating properties of MheI may also have value in the development of herbicide-resistant food or fibre crops.

In respect of free-enzyme contamination of wastewater, we note that ZimA has already been trialled successfully in decontaminating millimolar concentrations of carbendazim in rinsates from commercial potato processing operations (Pandey et al. 2010). High concentrations of other fungicides or insecticides against which ZimA/Mhe1 has activity could also occur in the post-harvest processing wastes of other horticultural operations (Felsot et al. 2003, Damalas et al. 2008, Scott et al. 2008, Galuszka et al. 2011, Delgado-Moreno et al. 2017), although, as noted, some enzyme engineering might be needed for cost-effective removal of some of those compounds from such waste streams. On the other hand, the phthalates and pesticides/herbicides encountered in many other waste waters of concern, such as from chemical production facilities and irrigated agriculture, are in the low nM–low µM range (Ritter 1990, Staples et al. 1997, Chang et al. 2007, Liang et al. 2008, Agarwal et al. 2015, Benjamin et al. 2015, Boll et al. 2020), and ZimA could be appropriate as is for many such situations.

The levels of activity required for enzymes deployed in phytoremediation are substantially lower than those needed in free-enzyme bioremediation because the enzymes can be produced semi-continuously by the host plant and have much longer time frames in which to act (Macek et al. 2008). The *mheI* gene may therefore be appropriate as is for phytoremediation trials against industrial chemicals without herbicidal activity, e.g. the phthalates. However, *in planta* applications directed at herbicides, including in herbicide-resistant crops, could clearly be more problematic. Notably MheI is secreted naturally by SG-4 G and is effective extracellularly in our assays, but different secretion machinery would be needed for *in planta* applications.

In conclusion, we find that ZimA/MheI has potential as a versatile resource for the bioremediation of a range of problematic compounds in various contamination situations. We have only tested a small number of the many carbamate and anilide pesticides and herbicides that are used in agriculture, so its substrate range is likely to be significantly wider than so far demonstrated. It will likely need optimization, including by modern enzyme biotechnology methods, for some substrates and applications, but for others, it may already be a relatively cost-effective option for bioremediation.

## Data sharing

Data sharing is not applicable to this article as no datasets were generated or analysed during the current study.

## Supplementary Material

fnad085_Supplemental_File
