# Supplementary material for: Wide substrate range for a candidate bioremediation enzyme isolated from Nocardioides sp. strain SG-4 G
Source: FEMS Microbiol Lett. 2023 Sep 2;370:fnad085. doi: 10.1093/femsle/fnad085 (PMC10501498; doi:10.1093/femsle/fnad085)
Supplement: fnad085_Supplemental_File [file fnad085_supplemental_file.docx]

**Unusually wide substrate range for a candidate bioremediation enzyme isolated from *Nocardioides* sp. strain SG-4G**

Kishore K Krishnani^1,2^, John G. Oakeshott^1,3^ and Gunjan Pandey^1*^

^1^CSIRO Land and Water, GPO Box 1700, Canberra, ACT 2601, Australia

^2^ICAR-Central Institute of Fisheries Education, Versova, Andheri (W), Mumbai-400061, India

^3^Applied BioSciences, Macquarie University, North Ryde, NSW 2109, Australia

Keywords: Plasticizers; Herbicides; Bioremediation enzyme; Biodegradation; Substrate specificity

*Corresponding authors

Gunjan Pandey

Email: [gunjan.pandey@csiro.au](mailto:gunjan.pandey@csiro.au)

Tel: +61 2 6246 4244

**Table S1.** Assay conditions for the substrates tested.

| Chemical | Solvent (run time in min) | Flow rate (ml/min) | DAD (nm) |
| --- | --- | --- | --- |
|  |  |  |  |
| Dimethly phthalate | 70% Acetonitrile (12) | 1 | 270-290 |
| Dibutyl phthalate | 90% Acetonitrile (10) | 1 | 270-290 |
| Dioctyl phthalate | 90% Acetonitrile (31) | 1 | 220-240 |
| Propanil | 65% Acetonitrile (10) | 1 | 280-300 |
| Monalide | 60% Acetonitrile (14) | 1.2 | 240-260 |
| Chlorpropham | 60% methanol (15) | 1.50 | 210 |
| Methomyl | 50% Acetonitrile (10) | 1 | 230 |
| Metsulfuron-methyl | 48% Acetonitrile (19) | 1 | 225 |
| Carfentrazone-ethyl | 75% Acetonitrile (10) | 1 | 234-255 |
| Pyrazosulfuron-ethyl | 70% Acetonitrile (10) | 1 | 240 |
| Sulfosulfuron | 60% Acetonitrile (9) | 1 | 235 |
| Imazethapyr | 48% Acetonitrile (10) | 1 | 235 |
| Pendimethalin | 60% Acetonitrile (20) | 1 | 240 |

**Table S2.** Microorganisms from which enzymes have been reported to degrade the phthalates, anilides or carbamates found to be substrate for MheI here and in Pandey et al. (Pandey et al., 2010), or related compounds. Where known, kinetic data for some substrates are also shown. Note that only one of the four MheI isolates other than the one herein from SG-4G is shown because the rest were not biochemically characterized in detail. **^*^** Abbreviations under the table.

| Microorganism | Substrate(s) for bacteria | Enzyme | Substrate(s) for enzymes | Not substrate(s) for enzymes | References |
| --- | --- | --- | --- | --- | --- |
| *Nocardioides* sp. SG-4G | Amides (PN, ML)  Carbamate (CIPC, MBC)  Dialkyl phthalate (DMP) | MheI  (ACV42482) | Amides (PN, ML)  Carbamate (CIPC, MBC)  Dialkyl phthalates (DMP, DBP, DOT) | Carbamate (DM)  Monoalkyl phthalates (trace activity, MMP, MBP, MOP) | This study  (Pandey et al., 2010) |
| *Microcystis aeruginosa* PCC 7941 |  | MheI  (CCI05716) | Carbamate (MBC) |  | GenBank entry only  (CCI05716) |
| *Paracoccus huijuniae*  Strain FLN-7 | Amides (DFB, PN, DM)  Carbamate (CIPC) | AmpA  (AFC37599) | Amides (PN, AMP, PM), Carbamate (CIPC) and OP insecticides with amide bonds (DM, OM) {*K*_M_ 81.1-29.5 µM, kcat (37.6-49.2) sec^-1^}  Benzoylureas (DFB, HF) | Carbamates (CF, CBL)  Ureas (DU, LU, MM) | (Sun et al., 2013; Zhang et al., 2012) |
| *Sphingobium quisquiliarum* DC-2 | Amide (acetochlor metabolite CMEPA) | CmeH  (AGR84269) | Amides (CMEPA, PN)  Phenoxy ester (CP) |  | (Li et al., 2013) |
| *Sphingomonas* sp. DC-6. | Amide (DM) | DmhA  (AGC74206) | Amides DM {*K*_M_ 20 µM and kcat 1.2 s‑1}, AMP and PN) | Amides (DFB, PP, CIPC LU) | (Chen et al., 2016) |
| *Ochrobactrum* sp. PP-2 | Amide (PN) | Mah  (ANS81375) | Amides (AMP, PN) and carbamates (CIPC, PP)  {*K*_M_ 6.3-13.7 µM, Kcat 10.5-20.0 sec^-1^} | Ureas (DU, LU) | (Zhang et al., 2019) |
| *Sphingomonas* sp. Y57 | Amide (PN)  Ureas (IP, DU) | PrpH  (AEO21835) | Amide (PN) | Ureas (IP, DU) | (Zhang et al., 2011) |
| *Fusarium oxysporum* | NA | Inducible amidase(s) | Amides (PN {*K*_M_ 7.1 x10^‑5^ M}, several other anilides) | Urea compounds | (Reichel, 1991) |
| *Microbacterium* sp. PAE-1 | Dialkyl phthalates (DBP, DMP, DEP, DBP, DPP, BBP, DHP, DEHP and the corresponding monoalkyl phthalates). | DpeH  (AZP89727) | Dialkyl phthalates (DMP, DEP, DBP, DPP, BBP, DHP, DEHP) {*K*_M_ 4.1-96.4 µM; Kcat 1.7×106 - 8.4×104 sec-1} |  | (Lu et al., 2020) |
|  |  | MpeH  (ZP89726) | Monoalkyl phthalates (MMP, MEP, MBP, MPP, mBzP, MHP, MEHP) {*K*_M_ 15.8- 52.5 µM, Kcat 8.6 x 105 - 1.2 x 106 sec-1} |  |  |
| *Acinetobacter*  sp. M673 | Dialkyl phthalates (DBP, DMP, DEP, DPRP, DPP, DHP, DEHP, DNOP, DNP) | Dph  (AFK31309) | Dialkyl phthalates (DMP, DEP, DPRP, DBP, DPP, DHP) {*K*_M_ 0.75 - 4.2 µM; Kcat 7.6- 22.7 sec-1} |  | (Wu et al., 2013) |
| *Rhodococcus* sp. EG-5 | NA | EG-5 MehpH  (BAU22081) | Monoalkyl phthalates (MEHP, MHP, MBP, MEP) {*K*_M_ 85-150 µM; Vmax 8.7-37 µmol.min^-1^.mg^-1^} |  | (Iwata et al., 2016) |
| *Rhodococcus jostii* RHA1 | Dialkyl (DMP, DEP, DPRP, DHP, DEHP) and monoalkyl phthalates (MMP, MBP, MHP, MEHP) phthalates | PatE  (ABH00399) | Monoalkyl phthalates (MMP, MBP, MHP, MEHP) {Crude extract specific activity 9.1-15.3 µmol.min^-1^.mg^-1^} |  | (Hara et al., 2010) |
| *Bacillus velezensis* SYBC H47 | NA | BaCEs04  (QEO33334.1) | Dialkyl phthalates (DMP, DEP, DPRP, DBP) {Specific activity 11.9 - 41.8 mmol min^-1^.mg^-1^} |  | (Huang et al., 2020) |
| *Rhodococcus* sp. 2G | Dialkyl phthalates (DMP, DEP, DBP, BBP, DOP, and DEHP) | Hyd  (AYW76486) | Dialkyl phthalates (DMP, DEP, DBP, DOP, DEHP, BBP, DINP) {*K*_M_ 0.53-1.6 mM; Kcat 34.2-175.4 s^-1^} |  | (Du et al., 2021) |
| Metagenome | NA | DphB  (AGY55960) | Dialkyl phthalates (DPrP, DBP, DPP {*K*_M_ 0.27-0.56 mM; Kcat 40.7-46.4 sec^-1^}, DMP, DEP, DHP, DHpP) | Dialkyl phthalates (alkyl chain <3 and >6) | (Jiao et al., 2013) |
| Metagenome | NA | EstJ  (QCQ29100) | Dialkyl and monoalkyl phthalates (DMP, DEP, DPRP, DBP, DPP, DHP, MMP, MEP, MBP, MHP) {*K*_M_ 0.4 - 3.6 mM; Kcat 9.4- 28.3 sec^-1^} |  | (Qiu et al., 2020b) |
| Metagenome | NA | EstYZ5  (QWT77157) | Dialkyl phthalate (DEHP {kcat/*K*_M_ 1272 mM^-1^. sec^-1^}) |  | (Yan et al., 2021) |
| Metagenome | NA | XtjR8  (QFQ13832) | Monoalkayl and dialkayl phthalates (MBP, DMP, DEP, DPRP, DBP, DPP, DHP) {*K*_M_ 0.86-2.3 mM; Kcat 12.45-36.5 sec^-1^} |  | (Qiu et al., 2020a) |

^*^Abbreviations: 2-chloro-N-(2-methyl-6-ethylphenyl)acetamide (CMEPA), 4-acetaminophenol (AMP), benzyl butyl phthalate (BBP), Carbaryl (CBL), carbendazim (MBC), Carbofuran (CF), chlorpropham (CIPC), clodinafop-propargyl (CP), di(2-ethylhexyl) phthalate (DEHP), dibutyl phthalate (DBP), Diethyl phthalate (DEP), diethylhexyl phthalate (DEHP), diflubenzuron (DFB), diheptyl phthalate (DHPP), dihexyl phthalate (DHP), dimethoate (DM), dimethyl phthalate (DMP), dinonyl phthalate (DNP), dioctyl Phthalate (DNOP), dipentyl phthalate (DPP), dipropyl phthalate (DPRP), diuron (DU), fenoxaprop-p-ethyl (FpE), hexaflumuron (HF), isoproturon (IP), linuron (LU), monalide (ML), mono-(2-ethylhexyl) phthalate (MEHP), monobenzyl phthalate (mBzP), monobutyl phthalate (MBP), monoethyl phthalate (MEP), monoheptyl phthalate (MHpP), monohexyl phthalate (MHP), monomethyl phthalate (MMP), monopentyl phthalate (MPP), monopropyl phthalate (MPrP), omethoate (OM), organophosphate (OP), propanil (PN), propham (PP)

**Figure S1.** Production of monomethyl phthalate from dimethyl phthalate by ZimA as seen in liquid chromatography (A) and LC-MS/TOF spectra of monomethyl phthalate (B) and dimethyl phthalate (C).


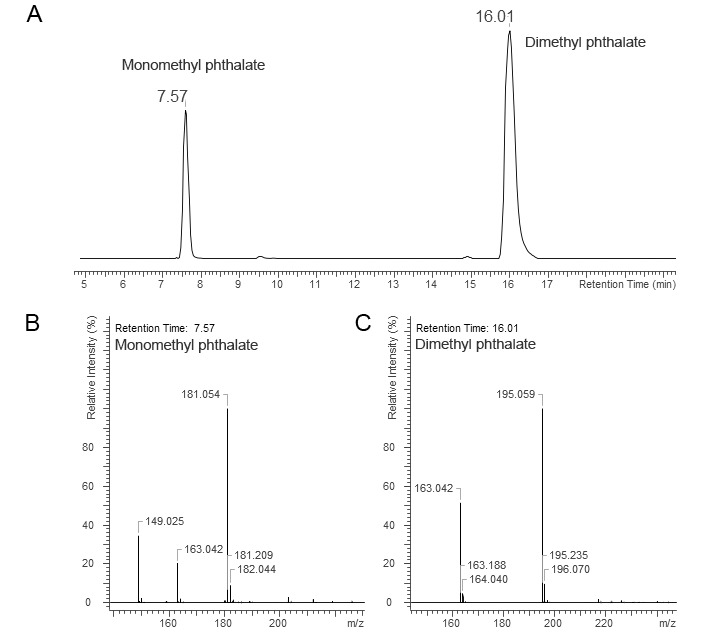


**Figure S2.** Stability of dibutyl phthalate in control (A) and production of monobutyl phthalate from dibutyl phthalate by ZimA (B) as seen in liquid chromatography and LC-MS/TOF spectra of dibutyl phthalate (C) and monobutyl phthalate (D).


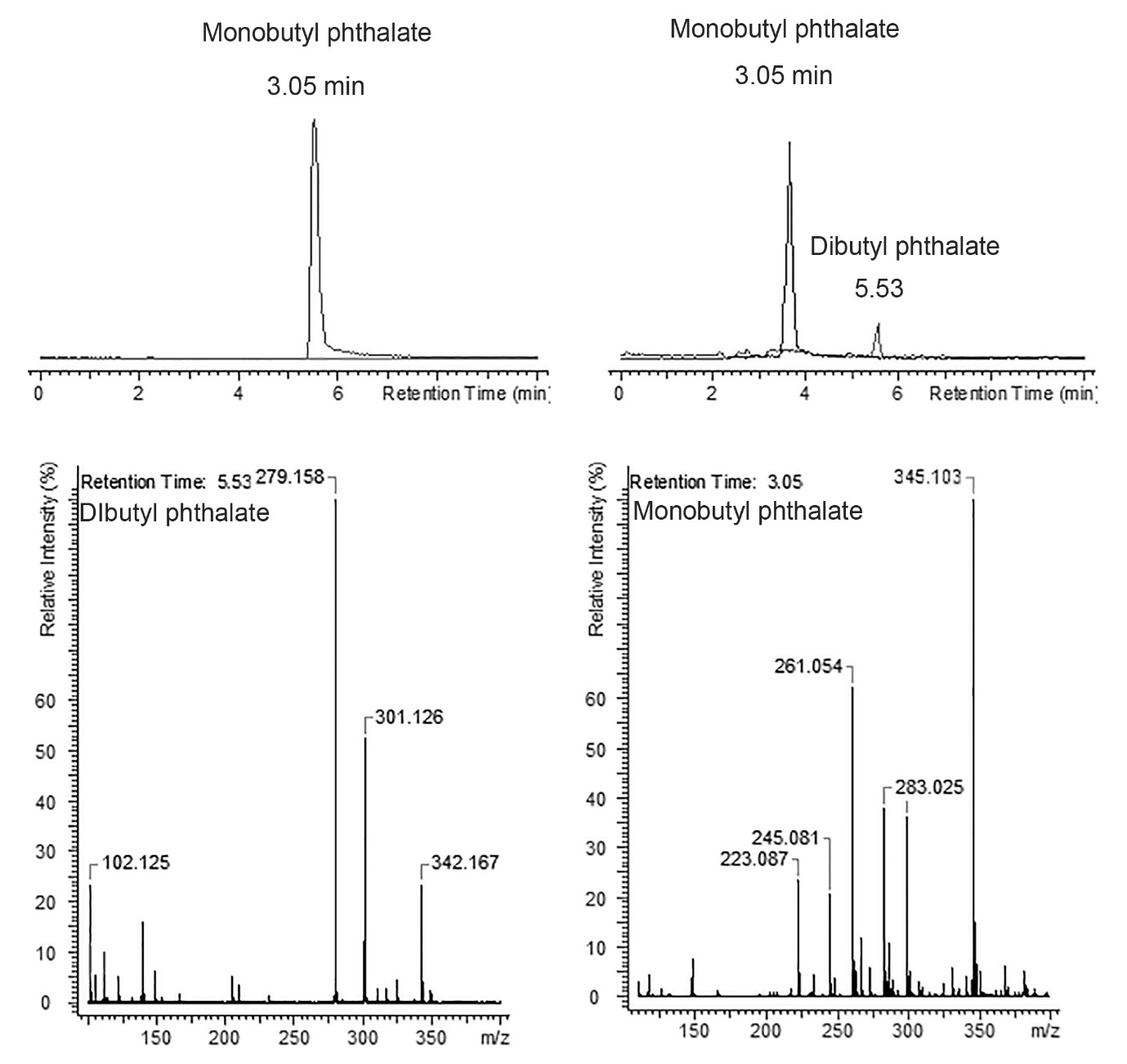


**Figure S3.** Stability of dioctayl phthalate in control (A) as seen in liquid chromatography and it’s LC-MS/TOF spectra (B). Complete transformation of dioctyl phthalate to monooctyl phthalate (C) as seen in liquid chromatography and LC-MS/TOF spectra of the latter (D).


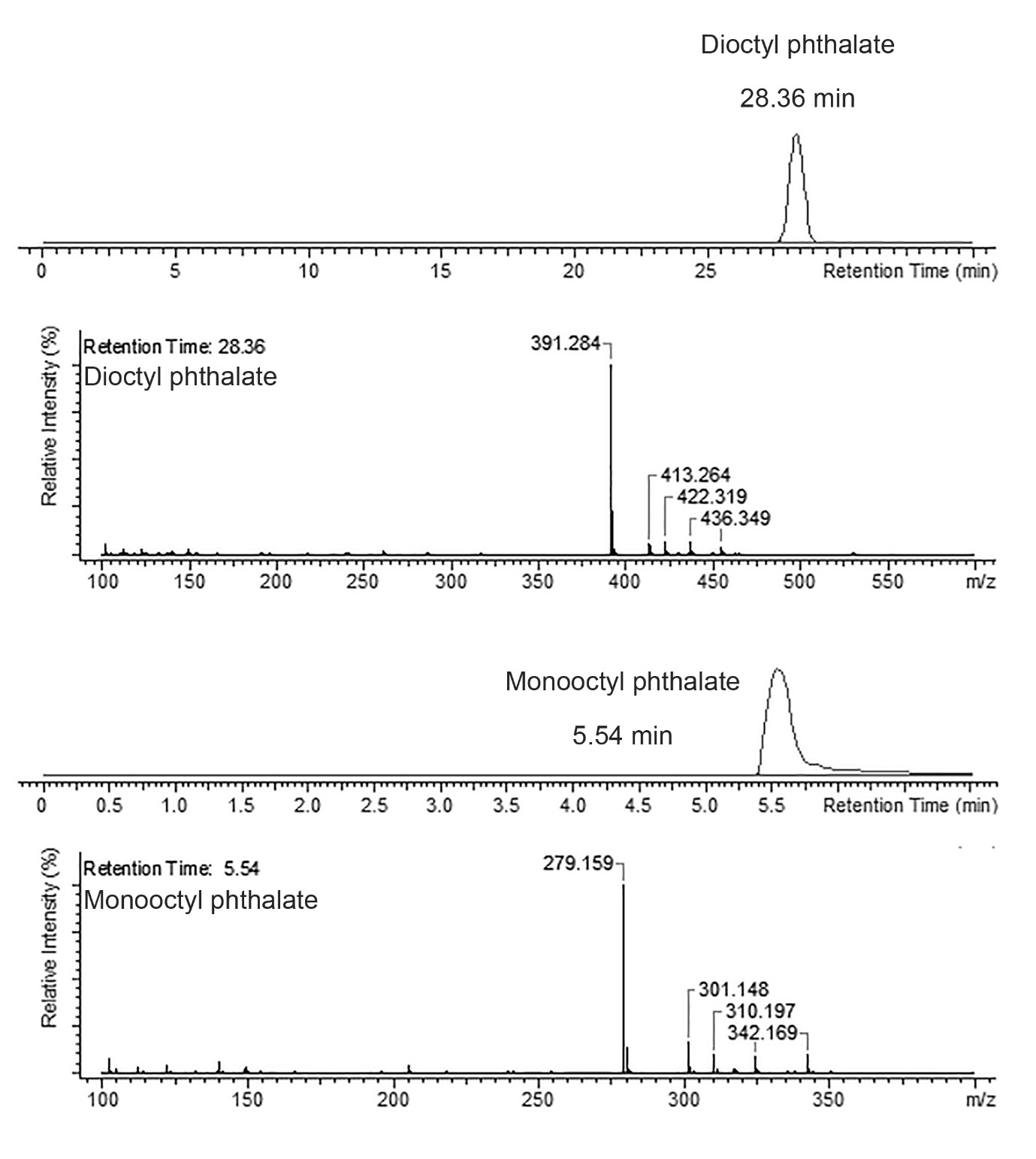


**Figure S4.** Stability of propanil in control (A) and its near complete degradation to 3,4-dichloroaniline by ZimA (B) as seen in liquid chromatography. LC-MS/TOF spectra of propanil (C) and its transformation product 3,4-dichloroaniline (D).

**
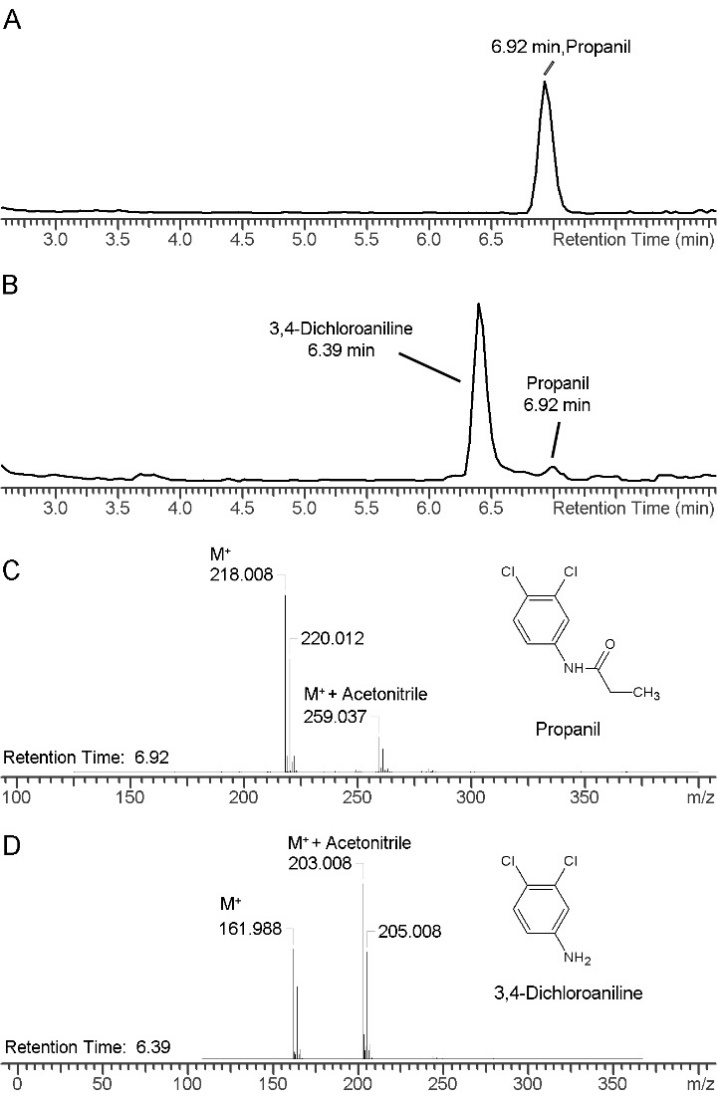
**

**Figure S5.** Plots of the amounts of propanil degraded in the first and second 12 h of a 24 h incubation with 800 ppm ZimA against the starting concentrations of the substrate. The raw data are the same as shown in Figure 4A.


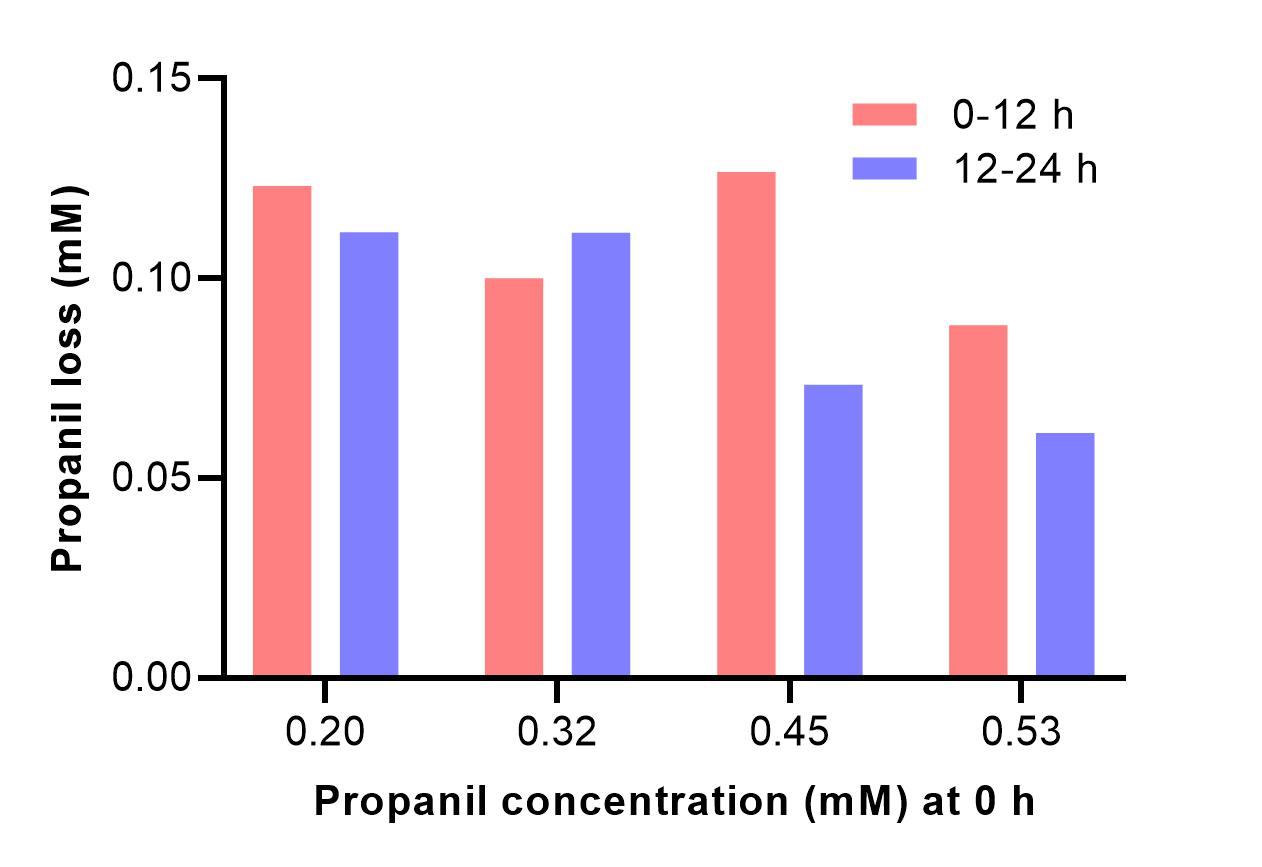


**Figure S6.** Production of 4-chloroaniline from monalide by ZimA as seen in liquid chromatography (A) and LC-MS/TOF spectra of monalide (B) and 4-chloroaniline (C). A small peak assumed to be the isomer impurity is also shown in Panel A.


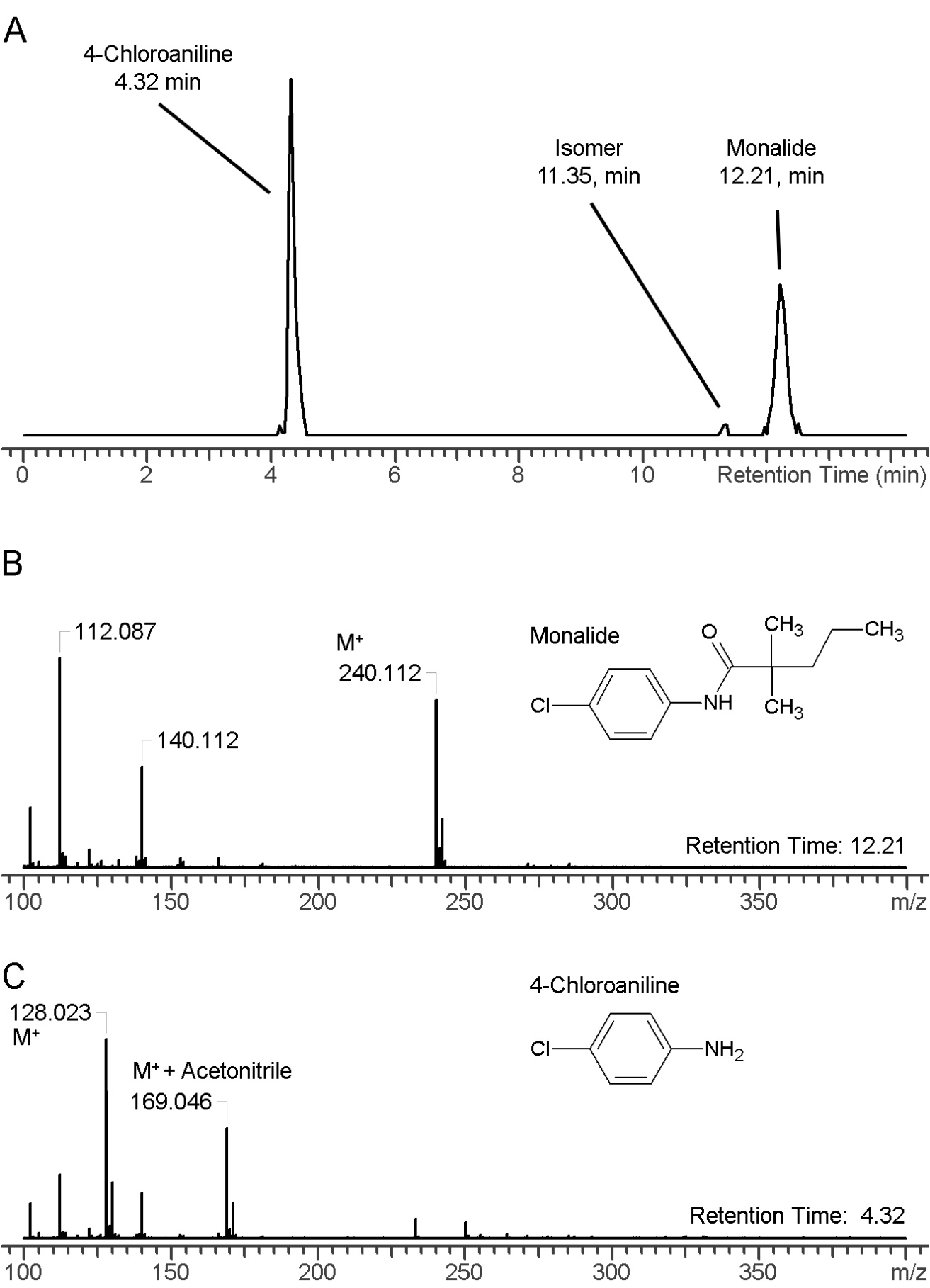


**Figure S7.** Comparison of the degradation kinetics of various concentrations of monalide and the putative isomer impurity by 800 ppm ZimA. The data for monalide are as per Figure 4B.


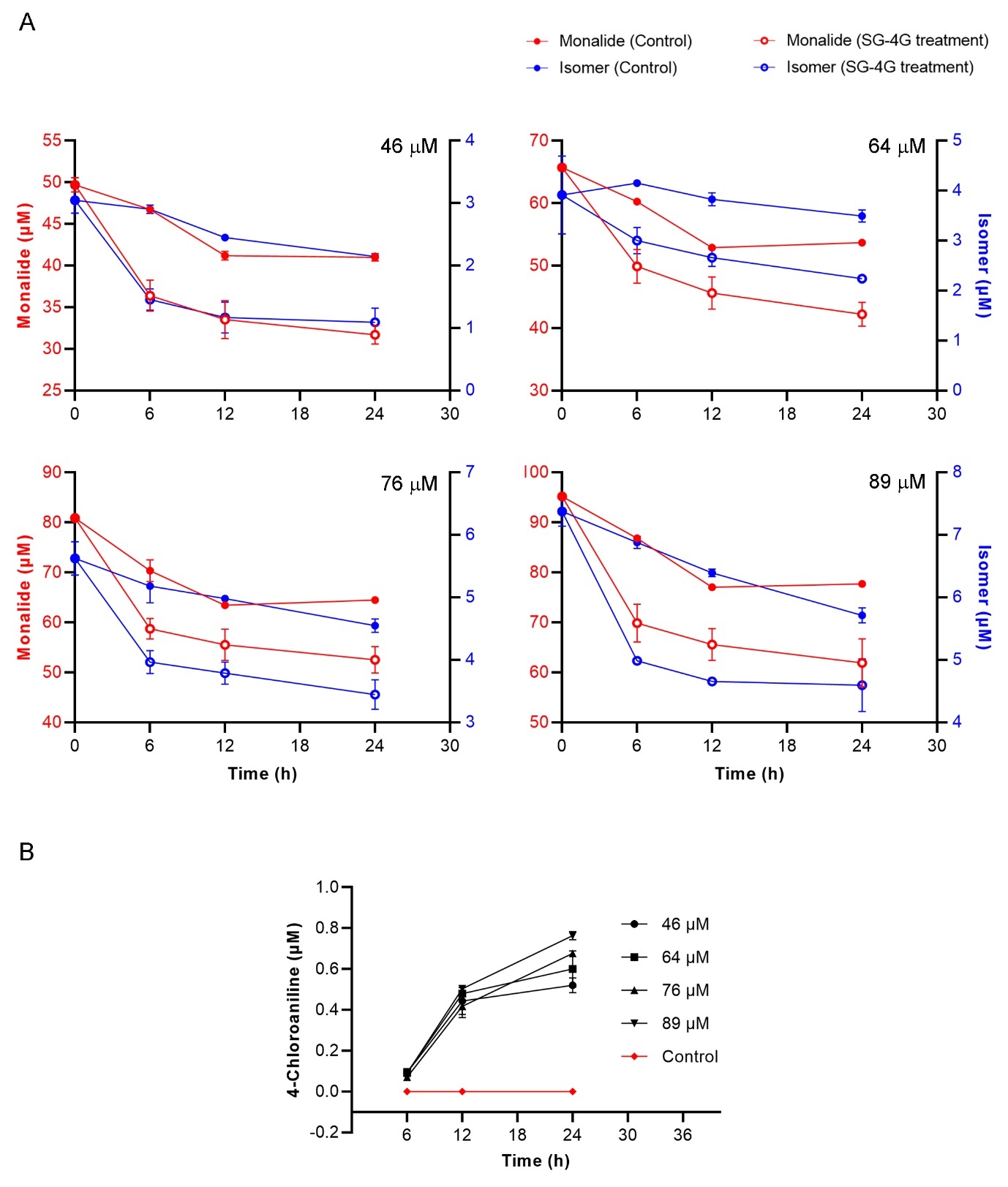


**Figure S8.**  Stability of chlorpropham in control (A) and its degradation to 3-chloroaniline by 100 ppm ZimA (B) as seen in liquid chromatography. LC-MS/TOF spectra of carbendazim (C) and its transformation product 3-chloroaniline (D).


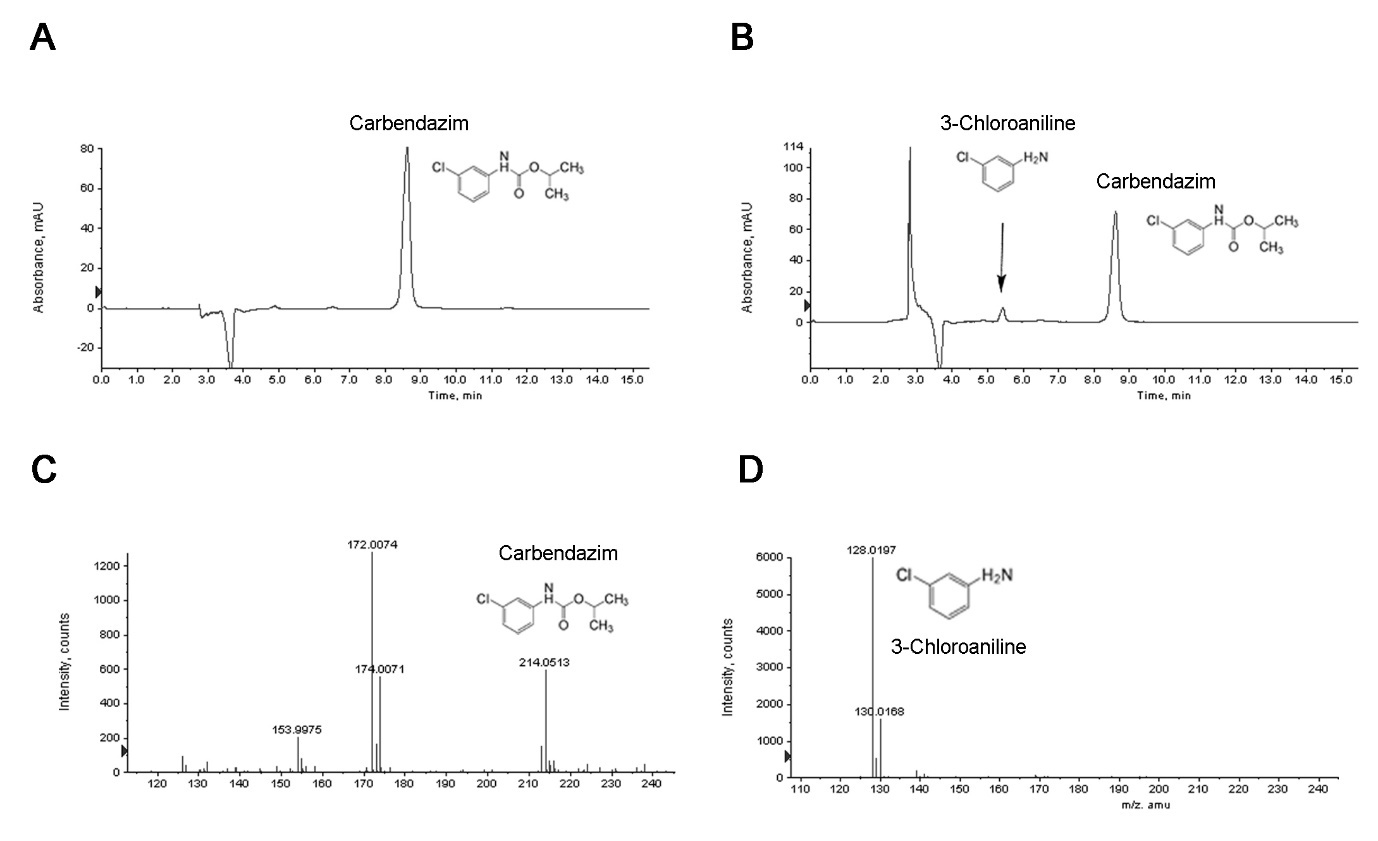


**Figure S9.** Mass spectra showing the transformation of carfentrazone-ethyl to carfentrazone by ZimA.


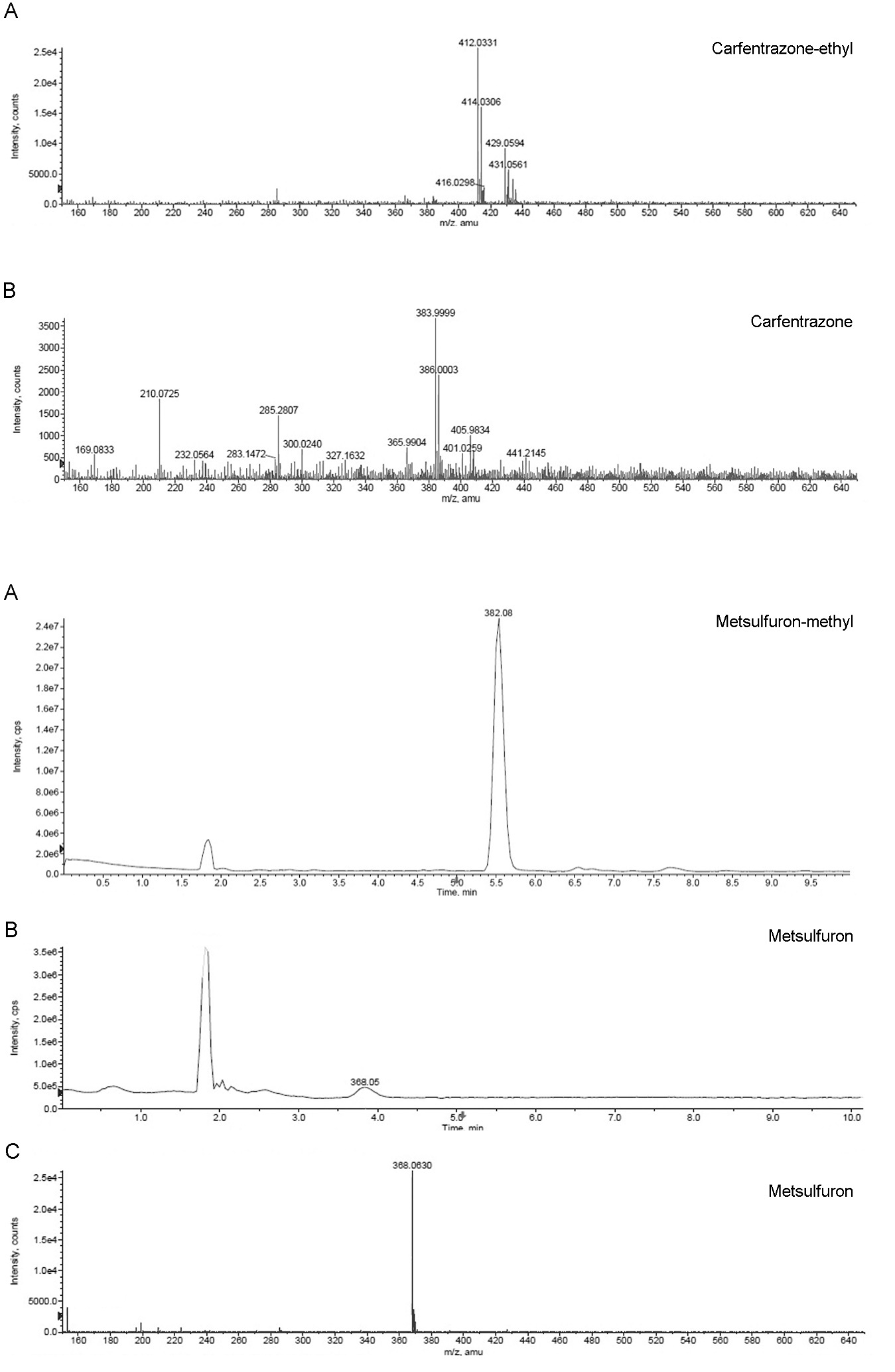


**Figure S10.** Liquid chromatography (A, B) and LC-MS/TOF (C) showing the conversion of metsulfuron-methyl to metsulfuron by ZimA


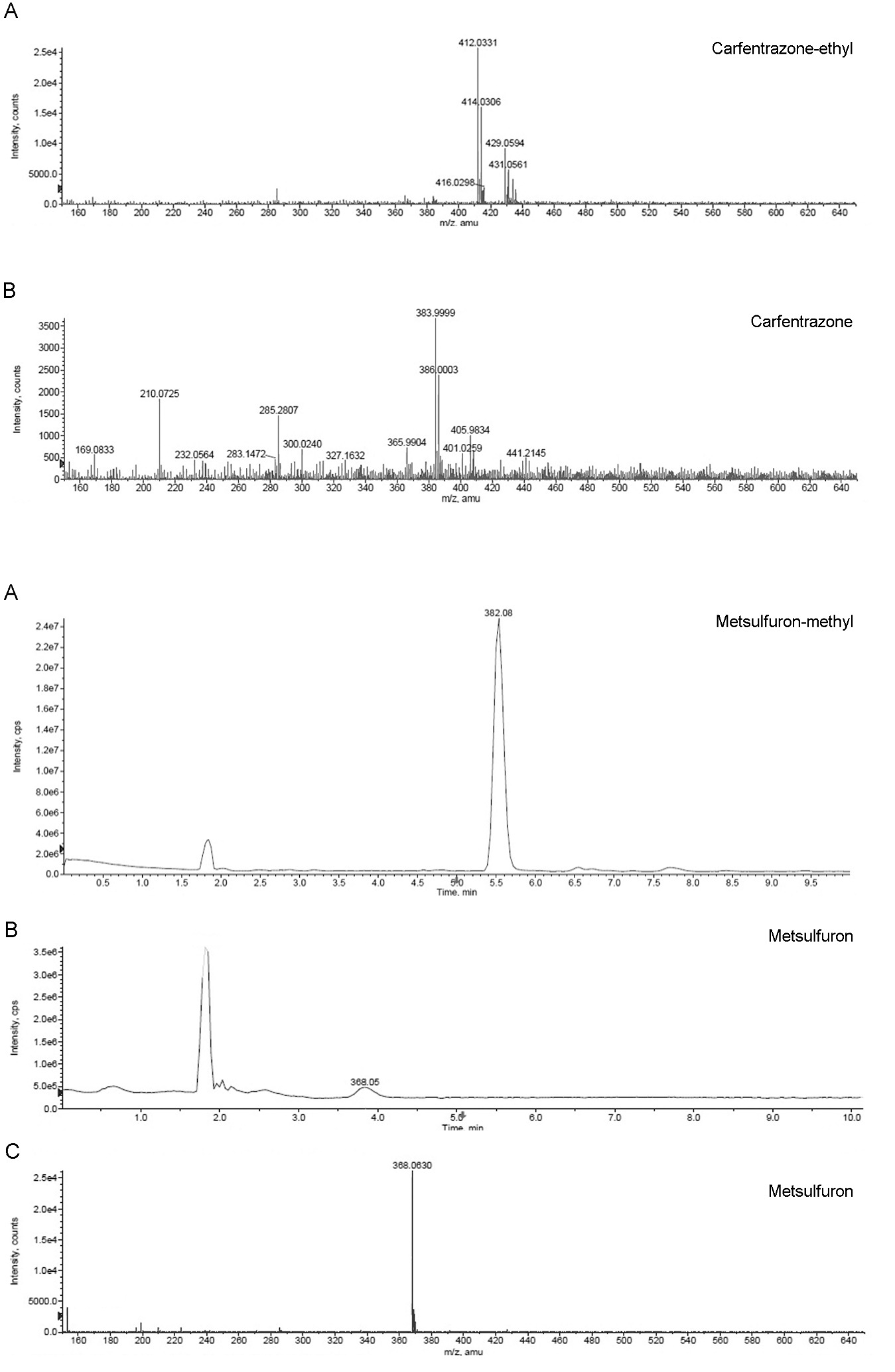


**References**

Chen, Q., Chen, K., Ni, H., Zhuang, W., Wang, H., Zhu, J., He, Q., He, J., 2016. A novel amidohydrolase (DmhA) from *Sphingomonas* sp. that can hydrolyze the organophosphorus pesticide dimethoate to dimethoate carboxylic acid and methylamine. Biotechnol Lett 38(4), 703-710. <https://doi.org/10.1007/s10529-015-2027-6>.

Du, H., Hu, R.W., Zhao, H.M., Huang, H.B., Xiang, L., Liu, B.L., Feng, N.X., Li, H., Li, Y.W., Cai, Q.Y., Mo, C.H., 2021. Mechanistic insight into esterase-catalyzed hydrolysis of phthalate esters (PAEs) based on integrated multi-spectroscopic analyses and docking simulation. J Hazard Mater 408, 124901. <https://doi.org/10.1016/j.jhazmat.2020.124901>.

Hara, H., Stewart, G.R., Mohn, W.W., 2010. Involvement of a novel ABC transporter and monoalkyl phthalate ester hydrolase in phthalate ester catabolism by *Rhodococcus jostii* RHA1. Appl Environ Microbiol 76(5), 1516-1523. <https://doi.org/10.1128/AEM.02621-09>.

Huang, L., Meng, D., Tian, Q., Yang, S., Deng, H., Guan, Z., Cai, Y., Liao, X., 2020. Characterization of a novel carboxylesterase from *Bacillus velezensis* SYBC H47 and its application in degradation of phthalate esters. J Biosci Bioeng 129(5), 588-594. <https://doi.org/10.1016/j.jbiosc.2019.11.002>.

Iwata, M., Imaoka, T., Nishiyama, T., Fujii, T., 2016. Re-characterization of mono-2-ethylhexyl phthalate hydrolase belonging to the serine hydrolase family. J Biosci Bioeng 122(2), 140-145. <https://doi.org/10.1016/j.jbiosc.2016.01.008>.

Jiao, Y., Chen, X., Wang, X., Liao, X., Xiao, L., Miao, A., Wu, J., Yang, L., 2013. Identification and characterization of a cold-active phthalate esters hydrolase by screening a metagenomic library derived from biofilms of a wastewater treatment plant. PLoS One 8(10), e75977. <https://doi.org/10.1371/journal.pone.0075977>.

Li, Y., Chen, Q., Wang, C.H., Cai, S., He, J., Huang, X., Li, S.P., 2013. Degradation of acetochlor by consortium of two bacterial strains and cloning of a novel amidase gene involved in acetochlor-degrading pathway. Bioresour Technol 148, 628-631. <https://doi.org/10.1016/j.biortech.2013.09.038>.

Lu, M., Jiang, W., Gao, Q., Zhang, M., Hong, Q., 2020. Degradation of dibutyl phthalate (DBP) by a bacterial consortium and characterization of two novel esterases capable of hydrolyzing PAEs sequentially. Ecotoxicol Environ Saf 195, 110517. <https://doi.org/10.1016/j.ecoenv.2020.110517>.

Pandey, G., Dorrian, S.J., Russell, R.J., Brearley, C., Kotsonis, S., Oakeshott, J.G., 2010. Cloning and biochemical characterization of a novel carbendazim (methyl-1H-benzimidazol-2-ylcarbamate)-hydrolyzing esterase from the newly isolated *Nocardioides* sp. strain SG-4G and its potential for use in enzymatic bioremediation. Appl Environ Microbiol 76(9), 2940-2945. <https://doi.org/10.1128/AEM.02990-09>.

Qiu, J., Yang, H., Yan, Z., Shi, Y., Zou, D., Ding, L., Shao, Y., Li, L., Khan, U., Sun, S., Xin, Z., 2020a. Characterization of XtjR8: A novel esterase with phthalate-hydrolyzing activity from a metagenomic library of lotus pond sludge. Int J Biol Macromol 164, 1510-1518. <https://doi.org/10.1016/j.ijbiomac.2020.07.317>.

Qiu, J., Zhang, Y., Shi, Y., Jiang, J., Wu, S., Li, L., Shao, Y., Xin, Z., 2020b. Identification and characterization of a novel phthalate-degrading hydrolase from a soil metagenomic library. Ecotoxicol Environ Saf 190, 110148. <https://doi.org/10.1016/j.ecoenv.2019.110148>.

Reichel, H.S., H. D.; Kaufman, D. D., 1991. Inducers, substrates, and inhibitors of a propanil-degrading amidase of *Fusarium oxysporum*. Pestic Biochem Phys 39(3), 240-250. <https://doi.org/https://doi.org/10.1016/0048-3575(91)90119-7>.

Sun, L.N., Zhang, J., Kwon, S.W., He, J., Zhou, S.G., Li, S.P., 2013. *Paracoccus huijuniae* sp. nov., an amide pesticide-degrading bacterium isolated from activated sludge of a wastewater biotreatment system. Int J Syst Evol Microbiol 63(Pt 3), 1132-1137. <https://doi.org/10.1099/ijs.0.044180-0>.

Wu, J., Liao, X., Yu, F., Wei, Z., Yang, L., 2013. Cloning of a dibutyl phthalate hydrolase gene from *Acinetobacter* sp. strain M673 and functional analysis of its expression product in Escherichia coli. Appl Microbiol Biotechnol 97(6), 2483-2491. <https://doi.org/10.1007/s00253-012-4232-8>.

Yan, Z., Ding, L., Zou, D., Qiu, J., Shao, Y., Sun, S., Li, L., Xin, Z., 2021. Characterization of a novel carboxylesterase with catalytic activity toward di(2-ethylhexyl) phthalate from a soil metagenomic library. Sci Total Environ 785, 147260. <https://doi.org/10.1016/j.scitotenv.2021.147260>.

Zhang, J., Sun, J.Q., Yuan, Q.Y., Li, C., Yan, X., Hong, Q., Li, S.P., 2011. Characterization of the propanil biodegradation pathway in *Sphingomonas* sp. Y57 and cloning of the propanil hydrolase gene prpH. J Hazard Mater 196, 412-419. <https://doi.org/10.1016/j.jhazmat.2011.09.040>.

Zhang, J., Yin, J.G., Hang, B.J., Cai, S., He, J., Zhou, S.G., Li, S.P., 2012. Cloning of a novel arylamidase gene from *Paracoccus* sp. strain FLN-7 that hydrolyzes amide pesticides. Appl Environ Microbiol 78(14), 4848-4855. <https://doi.org/10.1128/AEM.00320-12>.

Zhang, L., Hu, Q., Hang, P., Zhou, X., Jiang, J., 2019. Characterization of an arylamidase from a newly isolated propanil-transforming strain of *Ochrobactrum* sp. PP-2. Ecotoxicol Environ Saf 167, 122-129. <https://doi.org/10.1016/j.ecoenv.2018.09.127>.
